# The role of α-sheet structure in amyloidogenesis: characterization and implications

**DOI:** 10.1098/rsob.220261

**Published:** 2022-11-23

**Authors:** Tatum Prosswimmer, Valerie Daggett

**Affiliations:** ^1^ Molecular Engineering Program, University of Washington, Seattle, WA 98195-5013, USA; ^2^ Department of Bioengineering, University of Washington, Seattle, WA 98195-5013, USA

**Keywords:** alpha-sheet, amyloid, toxic oligomer

## Abstract

Amyloid diseases are linked to protein misfolding whereby the amyloidogenic protein undergoes a conformational change, aggregates and eventually forms amyloid fibrils. While the amyloid fibrils and plaques are hallmarks of these diseases, they typically form late in the disease process and do not correlate with disease. Instead, there is growing evidence that smaller, soluble toxic oligomers form prior and appear to be early triggers of the molecular pathology underlying these diseases. Nearly 20 years ago, we proposed the α-sheet hypothesis after discovering that the early conformational changes observed during atomistic molecular dynamics simulations involve the formation of a non-standard protein structure, α-sheet. Furthermore, we proposed that toxic oligomers contain α-sheet structure and that preferentially targeting this structure could neutralize the toxicity, prevent further aggregation and serve as the basis for early detection of disease. Here, we present the origin of the α-sheet hypothesis and describe α-sheet structure and the corresponding mechanisms of conversion. We discuss experimental studies demonstrating that both mammalian and bacterial amyloid systems form α-sheet oligomers before converting to conventional β-sheet fibrils. Furthermore, we show that the process can be inhibited with *de novo* designed α-sheet peptides complementary to the structure in the toxic oligomers.

## Graphical abstract

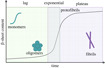

## 1. Introduction

Amyloidogenic proteins undergo conformational changes from their native structure, misfold and self-aggregate to form amyloid fibrils [1]. These self-aggregating proteins have been identified in both mammalian and bacterial species [1–3]. Mammalian amyloid proteins are associated with over 50 diseases, including Alzheimer's disease (AD), Parkinson's disease (PD) and Type 2 diabetes (T2D) [4,5]. Bacteria can also form amyloid fibrils using programmed machinery and incorporate the fibrils into their extracellular biofilms [2,3]. Biofilms protect the organism from the surrounding environment, including the host immune response and antibiotics [2,3]. Regardless of the function and native structure of an amyloidogenic protein, the corresponding fibrils form cross-β sheet structure [6]. Recent studies show that toxicity associated with amyloid diseases is due to the soluble oligomeric species rather than fibrillar plaques [7–11]. Furthermore, there is increasing evidence that both mammalian and bacterial toxic oligomers are comprised of a non-standard protein secondary structure known as α-sheet [9,12,13]. By targeting the α-sheet structure in the toxic oligomers, it may be possible to combat the pathology associated with human amyloid diseases, as well as infections associated with bacterial amyloid.

## 2. Amyloid proteins and their aggregation properties

**Figure 1.**
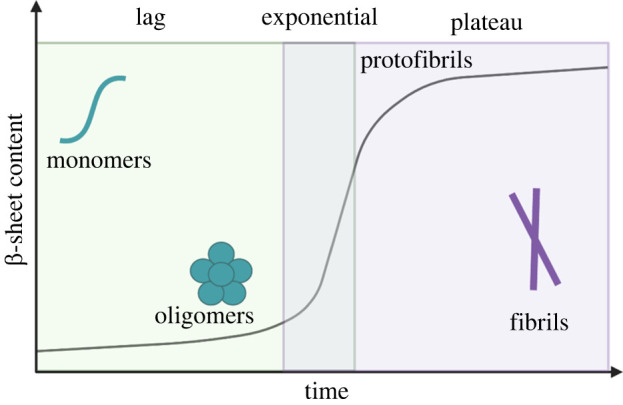
Amyloid aggregation pathway. The first step is known as the lag phase and is characterized by the formation of an amyloid-competent monomer and oligomerization. In the exponential phase, the amyloid protein changes structure and forms β-sheet protofibrils. Finally, the plateau phase describes the stage at which fibrils have rearranged to adopt highly ordered cross-β-sheet structure.

The amyloid aggregation pathway has been studied extensively, primarily in the context of human disease pathology. Aggregation occurs via a nucleation-dependent mechanism with three main phases of aggregation: lag, exponential and plateau (figure 1) [14]. The lag phase begins when a protein or peptide undergoes conformational changes to form an aggregation competent monomer [15]. This aggregation competent species alone has a low propensity for oligomerization, although small aggregates have the capacity to form over time [1,15]. The lag phase ends with the formation of an aggregation nucleus, the structure from which amyloid fibrils assemble [1,16]. The exponential phase is characterized by rapid oligomerization, resulting in the formation of fibrils composed of β-sheet structure [15]. The plateau phase describes the end of the aggregation pathway when fibrils, or plaques, are deposited in surrounding tissues [5].

Amyloid fibrils and plaques are the pathological hallmarks of amyloid diseases. As such, amyloid research has historically focused on the study and characterization of the fibrils, rather than the oligomers or the misfolded monomers. One of the most extensively studied amyloid systems is the amyloid-β peptide (Aβ), an intrinsically disordered peptide that is associated with AD [17]. Notably, extensive studies of Aβ and other amyloid fibrils show that the fibrils, or amyloid state, are not responsible for disease-related pathogenicity [8–10,17,18]. Instead, studies of Aβ oligomers indicate that the low molecular weight oligomers (approx. 8–70 kDa) are the toxic species linked to hippocampal long-term potentiation impairment, microglial activation and synaptic dysfunction [8,10,18]. Higher-molecular-weight oligomers, protofibrils and plaques display little to no cytotoxicity in biological assays [9,10]. In fact, plaque formation may serve a protective function by removing toxic oligomers from surrounding tissue [8].

The toxicity of low molecular weight soluble oligomers is not unique to Aβ and AD; studies show that the insoluble fibrils of many amyloid proteins are stable and non-toxic, while oligomers are the primary toxic species [8–10,19–22]. Islet amyloid polypeptide (IAPP), for example, is an intrinsically disordered peptide [23] whose amyloid formation is associated with T2D [24–26]. Electron microscopy experiments in which an aqueous solution of IAPP was applied to cultured islet cells demonstrated small IAPP oligomers disrupting the cell membranes, resulting in apoptosis [27]. This finding suggests that β-cell apoptosis associated with T2D is also likely caused by small, low molecular weight IAPP oligomers, rather than fibrils [27]. Given that oligomers are thought to be the trigger responsible for downstream pathology in amyloid-associated diseases, a deeper understanding of the structural characteristics of these low molecular weight species is necessary.

## 3. Cross-reactivity of A11 oligomer antibody suggests a conserved oligomeric structure

An attempt to probe the structural properties of Aβ oligomers by Glabe, Kayed and co-workers revealed the existence of a conserved conformation between amyloid oligomers, thereby introducing a novel tool through which to target and isolate this toxic species [19,28,29]. With knowledge of the structure of both Aβ monomers and mature fibrils, they sought to characterize the intermediary species which had previously eluded researchers by raising a polyclonal antibody, now known as A11, against molecular mimics of Aβ soluble oligomers. This was done to produce a molecule that targets the soluble toxic aggregates of Aβ with no specificity toward the monomeric or fibrillar forms [19,28,29]. While they successfully produced an antibody with specificity for Aβ oligomers, they found that A11 also binds to many oligomeric species, regardless of the amyloid protein's native structure or amino acid sequence [19,28,29]. A11 does not recognize the corresponding monomers or amyloid fibrils of these proteins, thereby exhibiting specificity for a particular structure or motif in toxic oligomers [19,28,29].

The cross-reactivity of the A11 antibody suggests that oligomers adopt a conserved structure, regardless of the amyloidogenic protein's native structure or sequence. In addition, A11 inhibits the toxicity of oligomers, including IAPP, Aβ, human insulin, lysozyme and more, when co-incubated and applied to live cells [19,28,29]. This suggests that not only do oligomers share a conserved conformation, they also share a conserved mechanism of toxicity that is inherently related to oligomeric structure [19,28,29]. Therefore, the production of the A11 antibody revealed that targeting oligomeric structure may serve as a strategic method to ameliorate toxicity and potentially combat amyloid-associated diseases.

## 4. Toxic oligomers are composed of α-sheet structure

Directly observing the conserved toxic oligomeric structure implicated by the cross-reactivity of the A11 antibody *in vivo* is challenging. This is due to the heterogeneous and interconverting mixture of misfolded monomers and oligomers that is present during the lag phase of aggregation [30]. Furthermore, elucidating the molecular mechanisms associated with amyloid formation is a challenging task because the process can span multiple orders of magnitude of time [31]. Molecular dynamics (MD) simulations serve as an alternative to experimental isolation; they can be used to predict the conformations sampled by a protein or peptide as it transitions from its native structure to an amyloid-competent monomer.

The Daggett lab conducted MD simulations of many structurally unrelated amyloidogenic proteins in order to study the conformational changes that occur over the course of aggregation. Proteins simulated included transthyretin (TTR), which has β-sandwich structure and is implicated in senile systemic amyloidosis and peripheral polyneuropathy [32–36]; the prion protein, a primarily helical protein involved in transmissible spongiform encephalopathies [37–44]; β_2_-microglobulin, a β-sheet protein implicated in hereditary renal amyloidosis [45]; lysozyme variants, small globular proteins involved in autosomal dominant hereditary amyloidosis [46]; polyglutamine repeats involved in Huntington's disease [47]; and superoxide dismutase-1, associated with amyotrophic lateral sclerosis [48,49]. Simulations were conducted under known amyloidogenic conditions to observe conformational changes that drive aggregation, including low pH and mutations associated with familial forms of the diseases [32–49].

A non-standard secondary structure, now referred to as α-sheet structure, was identified as an unfolding intermediate in simulations of each amyloid protein. We learned later that Linus Pauling and Robert Corey had predicted α-sheet structure in 1951, then referred to as ‘polar pleated sheet’ [50]. However, Pauling and Corey found that the structure was not an energetic minimum in their dihedral potential function, and it was therefore rejected. Until its independent ‘rediscovery’ in the Daggett lab, the structure was dismissed and considered to be a rare and alternative conformation to the energetically favourable β-sheet, which is correct for normal, native proteins. Following its observation in MD simulations conducted by the Daggett lab, α-sheet structure has been observed in a number of other MD simulations of amyloid proteins [51–56].

## 5. α-Sheet geometry and features of the structure

**Figure 2.**
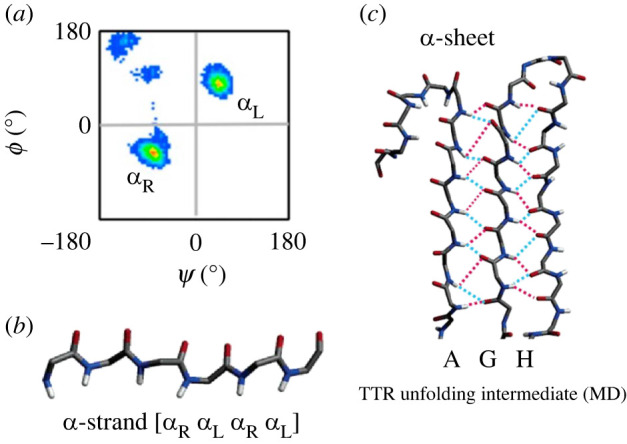
α-sheet structure. (*a*) Sequential amino acids in the α-strand conformation have backbone *Φ* and *Ψ* angles that alternately occupy the *α*_R_ and *α*_L_ regions of Ramachandran space. (*b*) The sequential alternation between *α*_R_ and *α*_L_ conformation results in the alignment of carbonyl groups on one side of the peptide backbone, and the alignment of the peptide's amide groups on the other side of the backbone. (*c*) Bifurcated hydrogen bonding between the A, G and H strands of TTR stabilize the peptide in α-sheet conformation. Reproduced from [12] and [32].

α-Sheet structure is stabilized by hydrogen bonding between individual α-strands, similar to the formation of standard β-sheet secondary structure [32,33,51,52,57,58]. Amino acids that form the strands are locally helical, occupying the left- (*α*_L_) and right-handed (*α*_R_) helical regions of Ramachandran space (figure 2*a*) [32,57–59]. Sequential alternation between *α*_L_ and *α*_R_ backbone (*Φ*, *Ψ*) dihedral angles results in the formation of an elongated strand (figure 2*b*) [32,52,57,59]. Sheets are formed with bifurcated hydrogen bonding between individual residues in adjacent strands (figure 2*c*) [32,57,60]. Bifurcated hydrogen bonding is also seen in α-helices, but not in β-sheet structures, and it can significantly increase structural stability [32,57]. α-Sheet is also unique in that the NH groups are aligned along one side of the sheet, while the carbonyl groups are located along the other [12,32,35,53,57]. The alignment of NH groups on one side of the sheet and CO groups on the opposite forms a strong molecular dipole across the sheet, which may assist in oligomerization through monomer–monomer interactions [32,57,61,62].

## 6. Mechanisms driving α-sheet formation

Beyond identifying a conserved oligomeric structure, MD simulations can also be used to determine the mechanism of structure formation in a particular amyloid system. Currently, the most well-studied α-sheet transition is that of TTR.

TTR exists in the body as a homotetramer and forms a β-sandwich structure composed of two β-sheets, referred to as the DAGH and CBEF sheets [33,35,63–67]. Both familial mutations and specific amyloidogenic conditions can lead to the destabilization of the tetramer, causing dissociation into an amyloid-competent monomer; notably, most cases are sporadic (WT) [33,35,68]. To further probe the mechanism of conversion from native β-sheet structure to α-sheet, 21 simulations were conducted on monomeric TTR under amyloidogenic conditions for 0.5 µs each [35]. Because both WT and mutant TTR monomers have been implicated in amyloid diseases, six pathogenic mutants (D18G, A36P, L58H, Y69H, L111M and V122I) in addition to the WT monomer were simulated in triplicate [35].

**Figure 3.**
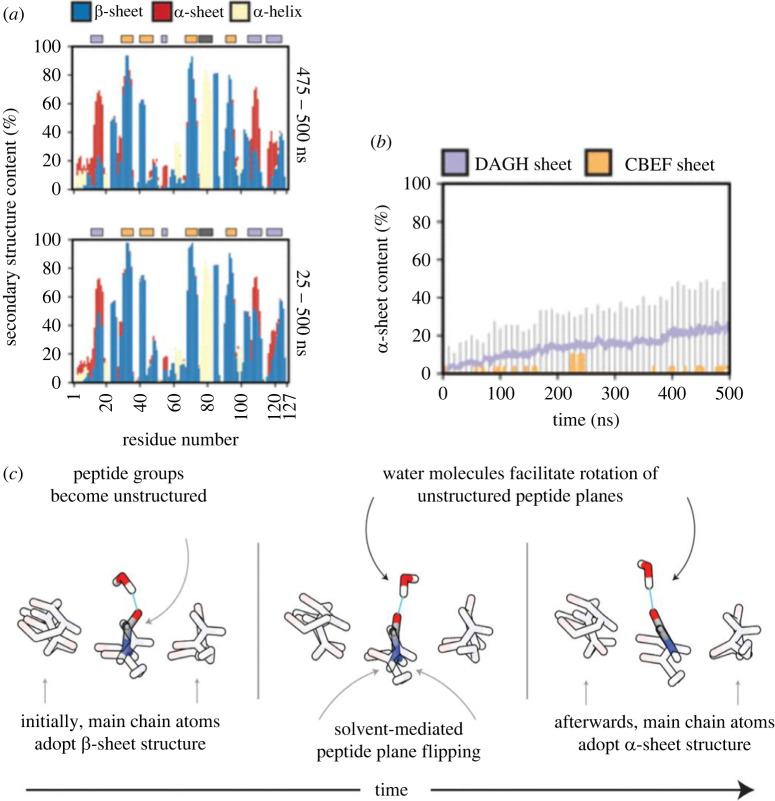
Conversion from native β-sheet to α-sheet structure in the DAGH sheet of TTR is observed in MD simulations of both wild-type and mutant TTR. (*a*) Secondary structure of each residue over the course of the simulation shows that residues 1–20 and 100–127 of the DAGH sheet (red) maintain α-sheet structure for approximately 60% of the total simulation time. (*b*) α-sheet content of the DAGH sheet increases linearly from 0 to 24% over the course of the simulations, while the CBEF sheet demonstrates little propensity toward α-sheet conversion. (*c*) Peptide plane flipping causes α-sheet conversion in the DAGH sheet of monomeric TTR. Once the peptide groups lose their native structure, water molecules participate in hydrogen bonding with the peptide's main-chain atoms to actively pull the backbone into the α-sheet conformation. Reproduced from [34].

A variety of analyses were used to determine both the extent and molecular mechanisms of α-sheet formation (figure 3*a*) [35]. The conversion of the DAGH native β-sheet structure to α-sheet structure was consistent among the seven simulated monomers, while the CBEF sheet largely maintained a stable β-sheet conformation (figure 3*b*) [35]. It was determined that peptide plane flipping causes α-sheet conversion in the DAGH sheet of both mutant and WT monomeric TTR (figure 3*c*) [35]. This change in secondary structure is preceded by three main events: distortion of main-chain geometries, referred to as ‘pleated main-chain geometry’, loss of the hydrogen bonding network between the main chain, and a reorientation of solvent-exposed side chain interaction networks (figure 3*c*) [35]. In addition to these phenomena, MD simulations revealed that hydrogen bonding employed by polar side chains and water molecules actively assists in pulling the peptide's backbone into α-sheet structure (figure 3*c*). Transitions not mediated by hydrogen bonding were driven by electrostatic repulsion of oxygen molecules present in the carbonyl groups in neighbouring strands [35].

This study outlines the molecular interactions that lead to the destabilization of the native β-sheet structure in TTR and subsequent conversion to α-sheet. The elucidation of these mechanisms further supports the α-sheet hypothesis and provides a blueprint through which to determine the molecular mechanisms involved in the conversion to α-sheet structure. The identification of the α-sheet conformation in TTR suggests that this structure may be responsible for the preferential oligomer binding behaviour exhibited by the A11 antibody. Indeed, synthetic *de novo* peptides that adopt stable α-sheet structure specifically bind to toxic TTR oligomers and inhibit amyloid formation [12], as discussed below, which supports the hypothesis that A11 recognizes and binds to α-sheet structure.

## 7. *De novo* designed synthetic model α-sheet peptides inhibit amyloid aggregation and toxicity

To experimentally probe the structure of soluble oligomers, *de novo* hairpin peptides were designed and engineered through MD simulations and produced by solid-phase peptide synthesis. These peptides were designed to adopt a conformation complementary to the non-standard α-sheet structure that was observed in MD simulations, and the designed peptides are themselves α-sheet. The designed peptides were hypothesized to selectively bind to the toxic oligomers of amyloid proteins regardless of the protein's native structure to facilitate isolation and characterization of the nonstandard structure [12].

The design process for these small, stable hairpins began with the computational development of a backbone template in an optimized α-sheet structure. The next step was to determine how to design the sequence to ensure alternation of sequential amino acids between *α*_R_ and *α*_L_ backbone conformations, which is the hallmark of α-sheet structure. To do this, the Structural Library of Intrinsic Residue Properties associated with the Dynameomics project was used to determine the propensities for various combinations of amino acids to occupy the desired regions of conformational space [69–75]. It was determined that sequential alternation between L- and D-amino acids would produce an extended sheet structure that mimics the α-sheet conformational signature observed in MD simulations of amyloid proteins containing all L-amino acids [12]. A variety of sequences were chosen according to these criteria and were used to engineer a library of *de novo* peptides predicted to adopt stable α-sheet structure. Finally, MD simulations of the α-sheet hairpins were conducted in conjunction with designed random coil and β-sheet control peptides to determine the stability of the *de novo* designs. The designs were then ranked, and the highest ranked designs were chemically synthesized and evaluated. The sequences corresponding to some of these designs and the controls can be found in table 1.

**Table 1.** The amino acid sequences of the *de novo* α-sheet peptides (AP). Residues that are denoted by a capital letter reference L-chirality amino acids, and D-chirality amino acid residues are depicted by lowercase letters. The underlined cysteine residues in AP407 refer to disulfide linkage sites. Each peptide has C-terminus amidation (shown as ‘NH2’), and ‘Ac’ refers to acetylation on the N-terminus.

	AP sequences
**AP5**	Ac-RGNwNeSkMNEYSGWmLmLtMGR-NH2
**AP90**	Ac-RGEmNlSwMNEYSGWtMnLkMGR-NH2
**AP193**	Ac-RGEmNyFwMNEYYGWtMnCkMGR-NH2
**AP401**	Ac-rGeMnLsWmneysGwTmNlKmGr-NH2
**AP407**	Ac-RGEmNlCwMNEYSGWcMnLkMGR-NH2
**AP421**	Ac-RGEcNlSwMNEYSGWtMnLkCGR-NH2
**P1**	Ac-KLKpLLTSENTL-NH2
**P90**	SWTWEpNKWTWK-NH2

## 8. Determination of spectroscopic signatures for α-sheet

The most stable designs as determined through MD were synthesized. As the structure is new and non-standard, it was necessary to determine the spectroscopic signature of α-sheet using the designed model compounds. The results from the model compounds were then used to determine whether amyloid intermediates and soluble oligomers contain α-sheet. Various techniques including circular dichroism (CD) spectroscopy, nuclear magnetic resonance (NMR) spectroscopy, Fourier transform infrared spectroscopy (FTIR) and microfluidic modulation spectroscopy in the infrared region (MMS-IR) were conducted on the α-sheet hairpin peptides in an effort to determine the spectroscopic signature of α-sheet secondary structure.

### 8.1. Circular dichroism spectroscopy

**Figure 4.**
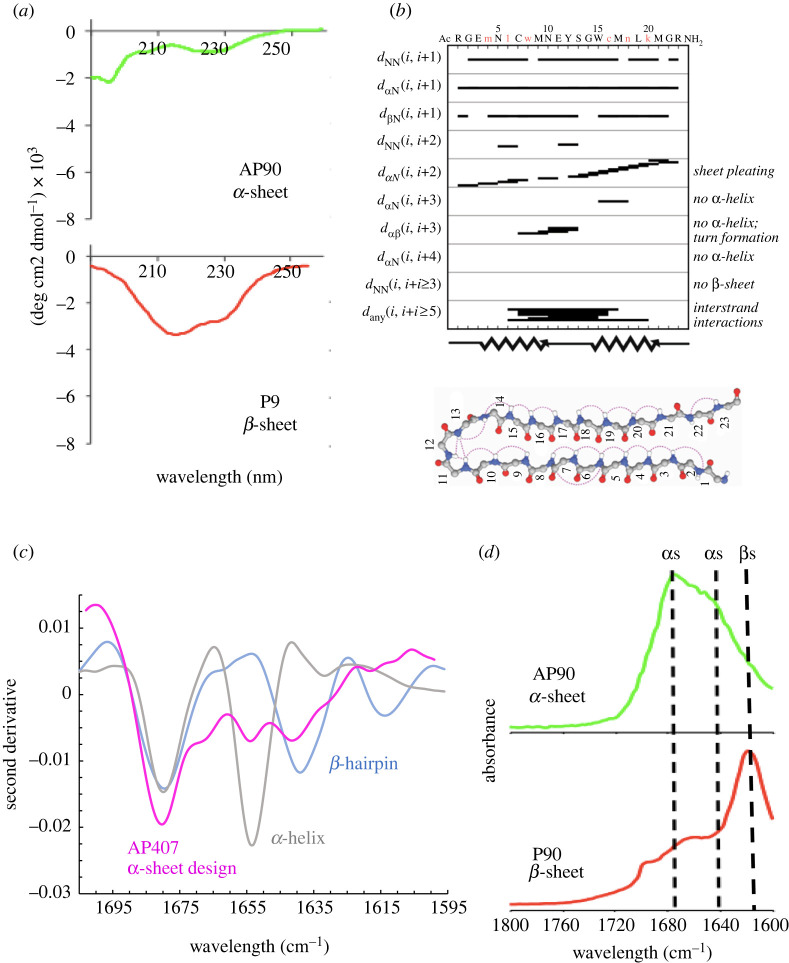
Spectroscopic characterization of *de novo* α-sheet peptides. (*a*) CD of an α-sheet peptide (AP90) displays relatively flat and featureless spectra due to the alternation of *α*_R_ and *α*_L_ residues, leading to cancellation of the CD signal. The dip around 200 nm is from the L-amino acids in the turn and at the ends of the hairpin. The all L-amino acid P90 peptide produced standard β-sheet spectra with a dip around 220 nm. AP90 and P90 have the same sequence, but AP90 contains six D-amino acids to template the α-sheet structure. (*b*) NMR of AP407 showed that the main-chain NOEs did not correlate to β-sheet or α-helical structure. The NMR ensemble of conformers determined for AP407, dominant conformer shown, confirms the two α-strands connected by a hairpin turn. (*c*) MMS-IR spectra of AP407, a β-hairpin control (P411) and an α-helix control (PSM*α*1) show that AP407 adopts a structure that is neither α-helical nor β-sheet. (*d*) Despite having the same amino acid sequence, AP90 and P90 produce distinct FTIR spectra, with P90 adopting β-sheet structure and AP90 displaying two bands associated with α-sheet, noting that the strength of the signals can change with 1675 cm^−1^ becoming less dominant in some AP designs [76]. Reproduced from [9] and [61].

CD spectroscopy is a technique that uses left- and right-handed circularly polarized light to evaluate the structure of chiral molecules. Due to the chirality of amide bonds, CD can effectively be used to study protein secondary structure. Because L- and D- amino acids absorb oppositely circularly polarized light, we predicted that CD of our *de novo* α-sheet peptides would produce relatively featureless spectra. As shown in figure 4*a*, the CD spectra of an α-sheet peptide, AP90, is indeed flat and primarily featureless with some random coil structure due to the influence of the tails and a hairpin turn composed of L-amino acids. In fact, AP401, which has inverse chirality of AP90, shows an inverse spectrum due to the dominance of D-amino acids in the hairpin turn and ends [13]. By contrast, the all L-amino acid version of AP90, P90, displayed a hallmark β-sheet spectrum with a minimum around 218 nm (figure 4*a*). Also of note is that AP90 and P90 have the same amino acid sequence, but AP90 contains 6 D-amino acids (6 of 23) to produce α-sheet, and P90 is comprised solely of L-amino acids. Despite having the same sequence, the peptides have different structures, solubilities and functions [61]. These results confirm that our *de novo* peptides adopt a structure that is distinct from random coil, β-sheet, and α-helical conformations.

### 8.2. Nuclear magnetic resonance structure of *de novo* designed α-sheet peptide

Homonuclear NMR spectroscopy was conducted on several of our *de novo* peptides [9,12]. One design in particular, AP407, was well characterized. The NMR studies of AP407 resulted in 455 nuclear Overhauser effect (NOE) crosspeaks between protons, a considerably large number considering the peptide length of only 23 residues (approx. 20 NOEs per residue). AP407 contains a disulfide bond linking the two α-strands, and this structural constraint likely contributed to the high number of NOEs. We expected to observe sequential *d*_NN_ NOEs along the backbone of our peptide, as predicted for α-sheet structure [12]. Further, α-sheet should not exhibit long-range *d*_NN_ or *d_α_*_N_ NOEs, which would indicate α-helical or β-sheet structure [12].

As expected, standard main-chain NOE patterns that are typical of α-helix and β-sheet structure were not observed (figure 4*b*) [9]. Instead, the NMR showed sequential H_N_-H_N_ NOEs that were predicted for α-sheet structure in addition to crosspeaks across the hairpin [9]. Further, the observed coupling constants suggest β-sheet structure while secondary chemical shifts suggest α-helical structure [9]. The combination of the coupling constant and secondary chemical shift results supports the extended sheet structure in which each residue is locally helical, which is precisely what we had hypothesized and designed into our *de novo* α-sheet peptides [9]. The NOEs were used to construct an NMR ensemble, with the dominant conformer of this small, dynamic peptide presented in figure 4*b*. NMR structural data have been presented and discussed in depth elsewhere [9], and they are available from the Biological Magnetic Resonance Data Bank (BMRB Entry 27873).

### 8.3. Microfluidic modulation spectroscopy

Next, we sought to further validate the presence of α-sheet structure in our *de novo* peptides through MMS-IR, which reports on amide I band absorption (1714 cm^−1^ to 1590 cm^−1^) [52]. Amide I band absorption is correlated with shifts in hydrogen bonding patterns and dipole–dipole interactions, and can therefore aid in determining protein secondary structure [77]. We conducted MMS-IR on a number of our *de novo* α-sheet peptides, including AP5, AP90, AP407 and AP421, in addition to β-sheet (P411) and α-helical (PSM*α*1) control peptides [9]. Comparing the MMS-IR spectra of the α-sheet, β-sheet and α-helical peptides confirmed that each of the three classes of peptides has distinct conformations and spectroscopic signatures, supporting and validating our original MD prediction of α-sheet structure (figure 4*c*).

### 8.4. Fourier transform infrared spectroscopy

FTIR was also employed on AP90 and its all L-amino acid structural isomer, P90, to help develop a spectroscopic signature for the non-standard α-sheet structure. Although these two peptides are composed of identical amino acid sequences, they produce very different FTIR spectra due to the presence of alternating L- and D- amino acids in AP90 (figure 4*d*) [12,61,76]. The aligned amide groups present in α-sheet structure (AP90) result in electrostatic interactions that are expected to produce strong FTIR signals, while these electrostatic interactions are not present in the structural isomer, P90 [12]. AP90 shows the strongest absorbance at 1675 and 1640 cm^−1^ [12,76], but the relative strength of these two bands depends on the sequence of the α-sheet peptides [76]. Conversely, the FTIR spectra of P90 is consistent with β-sheet structure with a strong absorbance at 1620 cm^−1^ (figure 4*d*) [61,76]. We can conclude from our FTIR experiments that the alternation of L- and D-chirality amino acids results in a unique and non-standard secondary structure that is spectroscopically distinct from its corresponding structural isomer.

## 9. α-Sheet in amyloid systems

### 9.1. Direct observation of α-sheet in amyloid proteins

As previously mentioned, isolation of a single conformer during the amyloid aggregation process is challenging due to the heterogeneous mixture of interconverting monomers, oligomers and protofibrils present prior to the deposition of stable fibrils [11]. However, aggregation conditions can be optimized to facilitate analysis of the various conformations sampled by an amyloidogenic peptide *in vitro* while maintaining a physiologically relevant environment [9,78,79]. The following studies were conducted using diligently engineered conditions to effectively lengthen the aggregation timeline and promote individual isolation of α-sheet and β-sheet containing species for spectroscopic characterization.

The engineered conditions include the use of more physiologically relevant buffers such as phosphate-buffered saline (PBS), rather than organic solvents, to modulate native aggregation [9,78,79]. Additionally, peptide concentration was carefully optimized for each system to ensure successive transitions between the lag, exponential and plateau phases of aggregation [9,78,79]. Using these engineered conditions, we can isolate essentially ‘pure’, or enriched, conformers for further experimental characterization, as described below.

#### 9.1.1. Circular dichroism spectroscopy

By engineering stable α-sheet peptides and subsequently characterizing the structure through a variety of spectroscopic techniques, we determined the spectroscopic signatures of α-sheet through which to observe and validate the existence of the structure in toxic oligomers in different amyloid systems. This spectroscopic signature has been used to directly probe α-sheet structure in the toxic oligomers of a number of mammalian amyloid proteins including IAPP and the 42-residue fragment of Aβ (Aβ42), in addition to multiple bacterial amyloid proteins, such as PSM*α*1, the amyloid protein in *Staphylococcus aureus (S. aureus)* biofilms, and CsgA, the amyloid protein in *E. coli* biofilms.

**Figure 5.**
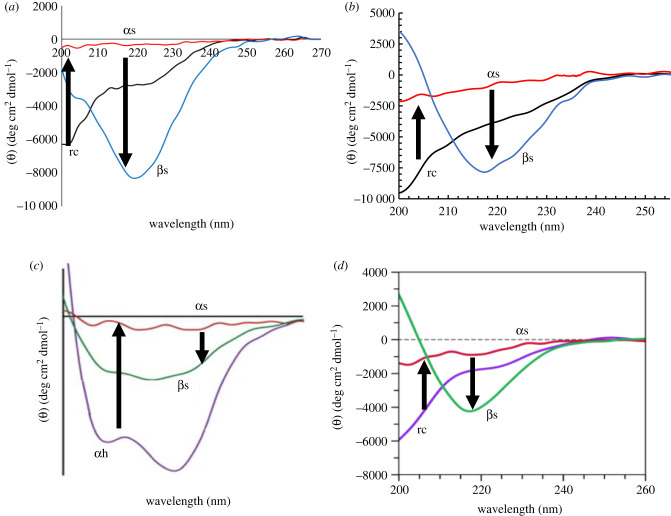
CD spectra of (*a*) islet amyloid polypeptide (IAPP), (*b*) Aβ42, (*c*) phenol soluble modulin a1 (PSM*α*1) from *S. aureus* and (*d*) CsgA from *E. coli* over the course of aggregation. IAPP, Aβ, and CsgA begin in a random coil state and PSM*α*1 is α-helical. Over time, each species produces a relatively flat and featureless CD spectrum, indicative of α-sheet conformation. At the end of aggregation, the amyloid species have β-sheet structure as shown by CD. All spectra are presented as MRE. Reproduced from [9,13,78,79].

CD spectroscopy was conducted on each of the aforementioned amyloid species over the course of aggregation to determine how secondary structure evolves throughout amyloidogenesis (figure 5*a–d*). At the beginning of incubation (*t* = 0 h), CD spectra of IAPP, Aβ, and CsgA indicated random coil structure, which is the expected structure for the monomeric species that exist at the beginning of aggregation for these peptides (figure 5*a,b,d*). By contrast, PSM*α*1 is natively α-helical, which is reflected in its CD spectrum at *t* = 0 h (figure 5*c*). Each of the four peptides have different aggregation kinetics, and therefore they form oligomers and adopt α-sheet structure after varying incubation times. While IAPP, Aβ, PSM*α*1 and CsgA form toxic oligomers at different times, each of these species produced a flat and relatively featureless CD spectrum at the end of their respective lag phase (figure 5*a–d*). Based on the spectroscopic signature derived from our *de novo* α-sheet peptides, we know that a flat, null spectrum is characteristic of α-sheet structure. Finally, during the plateau phase of aggregation, IAPP, Aβ, PSM*α*1 and CsgA all adopt β-sheet structure as shown by the strong CD signal around 218 nm (figure 5*a–d*). These CD studies allowed us to track secondary structure during aggregation, as well as observe a conserved secondary structure in four structurally and physiologically unrelated amyloid proteins.

#### 9.1.2. Microfluidic modulation spectroscopy

**Figure 6.**
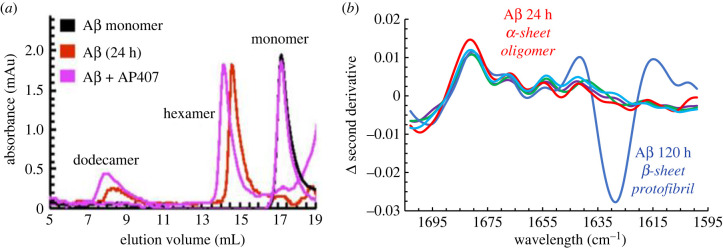
Selective binding of α-sheet peptide to toxic oligomers and spectroscopic identification of α-sheet in Aβ toxic oligomers. Aβ was incubated at 25°C for 24 h to obtain a sample enriched in toxic oligomers [9]. (*a*) AP407 binds specifically to oligomeric Aβ, as shown by SEC, depicted by the shift to higher molecular weight (lower elution volume) for the hexameric and dodecameric oligomers and the lack of binding to monomeric Aβ. (*b*) The subtraction of the second derivative of the MMS-IR spectrum of AP407 from each of the other signals, including three other α-sheet designs (shown as a band of three light blue toned lines), illustrates the similarities between the *de novo* α-sheet peptides. Furthermore, during the lag phase (24 h, in red) Aβ has a similar structure to the AP designs as shown by the second derivative of MMS-IR, confirming the presence of α-sheet conformation in the Aβ oligomers, as also observed by CD (figure 5*b*). In addition, toxic oligomeric Aβ displays a spectrum distinct from the other secondary structures, including the α-helical and monomeric β-hairpin model compounds as well as the non-toxic 120 h incubated Aβ protofibrils/fibrils with β-structure by CD (figure 5*b*) from the plateau of the ThT aggregation assay [9]. Reproduced from [9].

A combination of kinetic assays and size exclusion chromatography (SEC) data was used to inform MMS-IR studies of Aβ42 aggregates that were incubated for various durations. A toxic, late-lag phase Aβ sample (24 h incubation) was analysed by SEC and determined to be a low-molecular-weight oligomeric sample containing primarily hexamer aggregates with some dodecamer (figure 6*a*) [9]. This species was analysed by MMS-IR, and it was found that this oligomeric Aβ sample closely aligned with the secondary derivative of MMS-IR spectra of our *de novo* α-sheet peptide, AP407 [9]. Subtraction of the AP407 spectra from the spectra of each of the α-sheet and control peptides, as well as the Aβ samples, confirms that there is little to no variance between the second derivative of MMS-IR spectra of the 24 h Aβ sample and the *de novo* α-sheet peptides (figure 6*b*) [9]. By contrast, an Aβ sample that had a longer incubation period (120 h) was closely aligned with our β-sheet control, P411, and the shifted wavelength commonly associated with amyloid fibrils (figure 6*b*). This 120 h sample was shown by SEC to contain primarily higher molecular weight, β-sheet oligomers (figure 6*b*) [9]. This further confirms that amyloid peptides and proteins, including Aβ, form low molecular weight oligomers with α-sheet structure before transitioning to higher molecular weight aggregates that adopt β-sheet structure.

### 9.2. Indirect support of α-sheet in amyloid proteins

#### 9.2.1. Inhibition of amyloid formation by targeting α-sheet oligomers

**Figure 7.**
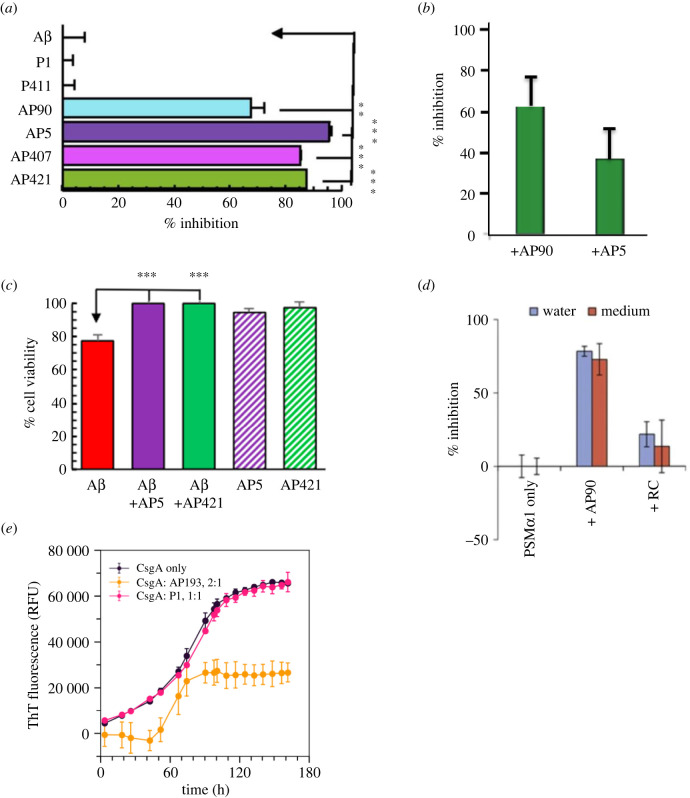
α-Sheet peptides inhibit amyloid formation and oligomeric toxicity. (*a*) Incubation of Aβ with excess (4 : 1) α-sheet peptide resulted in up to 96% inhibition, while control peptides (P1 and P411) had no effect on aggregation. (*b*) Incubation of TTR with excess (10 : 1) AP90 and AP5 resulted in 63% and 37% inhibition, respectively. (*c*) Exposure to oligomeric Aβ resulted in almost 20% loss of viability of SH-SY5Y cells. Incubation with AP5 and AP421 resulted in complete recovery of cell viability. AP5 and AP421 themselves were not toxic to the cells. (*d*) Incubation of PSM*α*1 with excess (4 : 1) AP90 resulted in 90% aggregation inhibition. The random coil control had little effect. (*e*) Incubation with excess CsgA (2 : 1) and AP193 resulted in approximately 60% inhibition, while the random coil control (P1) had no effect on aggregation. While the extent of inhibition differs among the various systems, α-sheet designs inhibit aggregation and toxicity in all systems independent of the sequence of the AP peptide and the sequence of the amyloid peptides/proteins. Statistical significance: *p < 0.05, **p < 0.01, and ***p < 0.001. Reproduced from [9], [13] and [78].

With the knowledge that binding the A11 antibody inhibits the toxicity of amyloid oligomers by recognizing a conserved conformation, we sought to determine whether our *de novo* peptides could selectively bind to and inhibit both the aggregation and the toxicity of these species. We tested a number of amyloid species, including both mammalian and bacterial proteins [3,9,12,61,76,78,79]. We found that our *de novo* α-sheet peptides significantly inhibited both the aggregation and toxicity of the amyloid proteins, while the control peptides had no effect on either the aggregation or the toxicity (figure 7*a–e*) [3,9,12,62,76,78,79].

SEC was employed to confirm that the observed inhibition with α-sheet peptides was due to the inhibition of amyloid formation through preferential binding to the oligomeric species with the same conformation, rather than through interactions with the corresponding random coil monomers [9]. Monomeric Aβ samples analysed both in the presence and in the absence of AP407 produced essentially identical SEC data, suggesting that the α-sheet peptide does not bind specifically to monomeric, random coil Aβ (figure 6*a*) [9]. By contrast, an oligomeric (24 h incubation) Aβ sample with AP407 showed an approximately 0.4 ml peak shift to a higher molecular weight with respect to the same Aβ sample alone (figure 6*a*) [9]. This indicates that AP407 binds preferentially to oligomeric Aβ, as hypothesized [9].

After confirming that *de novo* α-sheet peptides bind preferentially to α-sheet containing toxic oligomers, we used a Thioflavin T (ThT) assay to probe whether this binding could lead to inhibition of aggregation [3,9,12,76]. ThT is a fluorescent dye that is often used to track amyloid formation [80]. Because it binds to β-sheet rich structures, it can serve as a proxy for the extent of fibrillization [80]. Low ThT signals are emitted during the lag phase of aggregation because there is little to no β-sheet in solution. The ThT signal exponentially increases following the lag phase as the peptides rapidly oligomerize and form fibrils. Finally, the ThT signal plateaus once highly ordered β-sheet containing fibrils have formed.

The extent of amyloid inhibition by our α-sheet peptides was quantified by measuring the ThT signal both in the presence and in the absence of the α-sheet designs. The incubation of Aβ with excess (4 : 1) α-sheet peptide resulted in up to 96% aggregation inhibition as shown by the reduced ThT signal (figure 7*a*) [9]. Random coil (P1) and β-sheet (P411) control peptides had no significant effect on aggregation (figure 7*a*) [9]. Incubation of TTR with excess (10 : 1) AP5 and AP90 resulted in approximately 37% and 65% inhibition, respectively (figure 7*b*) [76]. As with the A11 antibody, α-sheet peptides also inhibit toxicity of amyloid oligomers [9,12,61,62]. The addition of α-sheet peptides (AP5 and AP421) to toxic Aβ oligomers immediately prior to plating with SH-SY5Y neuroblastoma cells resulted in complete recovery of cellular viability, thereby inhibiting oligomeric toxicity (figure 7*c*) [9]. The α-sheet peptides themselves were not toxic to the cells (figure 7*c*) [9].

α-Sheet peptides have also proved effective at inhibiting the aggregation of bacterial amyloid. PSM*α*1, the amyloid protein present in *S. aureus* biofilms, incubated with excess (4 : 1) AP90 resulted in 81% amyloid inhibition (figure 7*d*) [13]. Incubation of AP193 with excess CsgA (1 : 2), the amyloid protein that composes *E. coli* biofilms, resulted in approximately 50% inhibition, while incubation with equal (1 : 1) random coil control (P1) had no effect on aggregation (figure 7*e*) [78]. Combined with the SEC results, we conclude that aggregation inhibition is due to the binding of α-sheet peptides to α-sheet containing oligomers, thereby preventing subsequent fibrillization.

**Figure 8.**
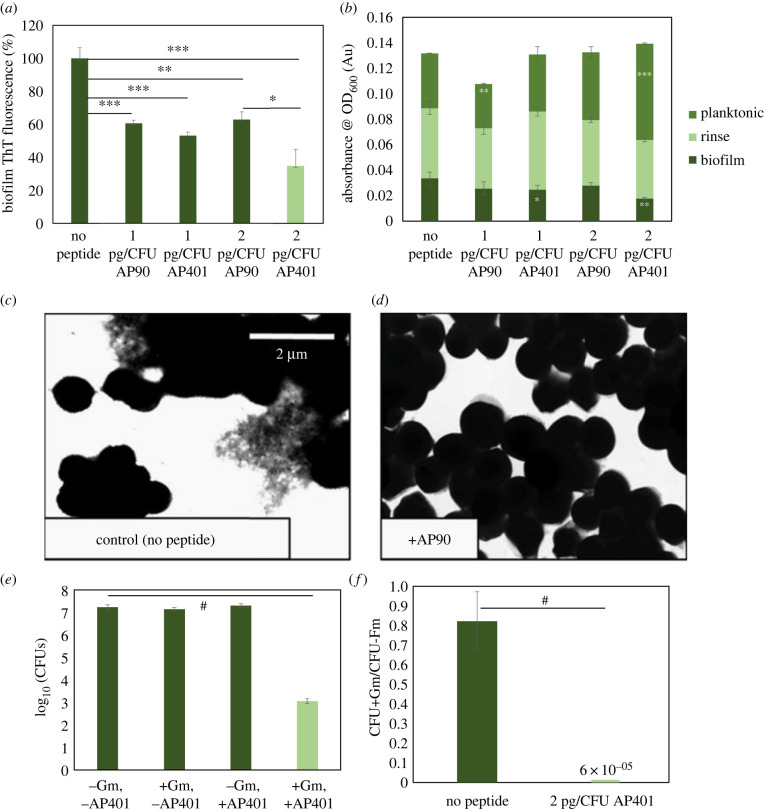
α-Sheet peptides inhibit biofilm amyloid content and cell density, leading to an increase in the susceptibility of the bacteria to antibiotics. (*a*) Incubation with 2 pg CFU^−1^ AP90 and AP401 resulted in 37% and 65% reduction of amyloid in *E. coli* biofilms, respectively, as measured by ThT. (*b*) AP90 and AP401 reduced the cell density of *E. coli* biofilms by 15% and 47%, respectively, as measured by OD_600_. Planktonic cell density increased 23% with AP90 and 75% with AP401, while the total cell density remained statistically unchanged. (*c*) PSMa1 amyloid fibrils are visible as deposits in spaces between cells of *S. aureus* SH1000 WT biofilms. (*d*) No extracellular matrices, or fibrils, were visible upon the addition of AP90. (*e*) Incubation of *E. coli* with 2 pg CFU^−1^ AP401 and 300 ug ml^−1^ gentamicin resulted in a reduction of CFUs by a factor of over 10^4^ with respect to the control conditions. (*f*) This CFU reduction corresponds to a 13 000× increase in susceptibility to gentamicin, calculated by comparing the ratio of CFUs following antibiotic administration, with and without the addition of AP401. CFU ratios are calculated based on cell density at the time of plating. Statistical significance: *p < 0.05, **p < 0.01, ***p < 0.001 and *^#^p*< 0.0001. Reproduced from [13] and [78].

Inhibition of bacterial amyloid can also be observed by comparing β-sheet content and cell density of biofilms grown in the presence and in absence of α-sheet peptides. For these studies, bacteria are grown in biofilm-forming conditions and assayed once the biofilm has matured. Planktonic, free-floating cells are removed following biofilm maturation and cell density is estimated through optical density measurements at 600 nm (OD_600_). The biofilm is then resuspended in ThT and measurements are taken both for ThT signal and OD_600_. These *in vivo* studies have been conducted on a number of both gram-positive and gram-negative bacteria, including *E. coli*, *P. aeruginosa*, *S. aureus* and *S. mutans* [13,78,81]. In each of these studies, incubation with α-sheet peptides resulted in a reduced ThT signal and decreased biofilm OD_600_ measurements [13,78,81]. For example, incubation of *E. coli* with 2 pg/colony forming unit (CFU) of AP90 and AP401 resulted in 37% and 65% reduction in biofilm amyloid content, respectively (figure 8*a*). Biofilm cell density, as measured by OD_600_, was reduced 15% and 47% when incubated with AP90 and AP401, respectively (figure 8*b*). Furthermore, OD_600_ measurements of the planktonic phase increase when grown in the presence of α-sheet peptides, indicating an increase in the number of free, non-biofilm-protected bacteria (figure 8*b*). We can therefore conclude that not only does exposure to α-sheet peptides inhibit bacterial amyloid formation, it also disrupts the biofilms, thereby causing more cells to remain in the planktonic phase rather being incorporated into the protective extracellular matrix that is the biofilm. It is also important to note that by comparing total OD_600_ measurements of the biofilm and planktonic phases, we can conclude that α-sheet peptides do not cause cell death, but rather inhibit amyloid aggregation and robust biofilm formation (figure 8*b*). This is crucial because if α-sheet peptides caused bacterial cell death, it would be possible for mutations to arise that would confer resistance to the peptides, as has occurred with antibiotics.

Bacterial biofilm inhibition with α-sheet peptides can also be observed *in vivo* through various imaging techniques. For example, *S. aureus*, *S. mutans* and *E. coli* form robust biofilms, but when these same bacteria are grown in the presence of α-sheet peptides, the biofilms are visibly less robust and the amyloid content drops dramatically, as illustrated in figures 8*c,d* for *S. aureus* [13,78,81]. This is in agreement with ThT and OD_600_ measurements that show that α-sheet peptides inhibit biofilm formation and force more cells to remain free-floating rather than be incorporated into the biofilm [13,78,81].

#### 9.2.2. Antibiotic susceptibility and α-sheet inhibitors of amyloidogenesis

Biofilms can significantly reduce the efficacy of antibiotics and can confer antibiotic resistance by providing structural support and allowing for communication between individual cells [82,83]. Targeting bacterial biofilms is one strategy through which to increase the susceptibility of bacteria to antibiotics without the added risk of increased resistance due to selective pressure. Having previously shown that incubation with α-sheet peptides significantly decreases biofilm amyloid content, weakens the biofilm, and reduces the number of cells present in and protected by the biofilm, we investigated whether this biofilm inhibition could lead to increased antibiotic susceptibility to resistant strains. Uropathogenic *E. coli* was used as our model system; we found that cells incorporated into mature *E. coli* biofilms were 13 000× more susceptible to gentamicin when grown in the presence of AP401 (figure 8*e,f*) [78]. Therefore, exposing *E. coli* to α-sheet peptides can render apparent antibiotic-resistant bacterial strains susceptible, and they may also aid in reducing antibiotic resistance, which has major implications on the future of targeting drug-resistant bacteria.

#### 9.2.3. Soluble oligomer binding assay for detection of α-sheet oligomers

**Figure 9.**
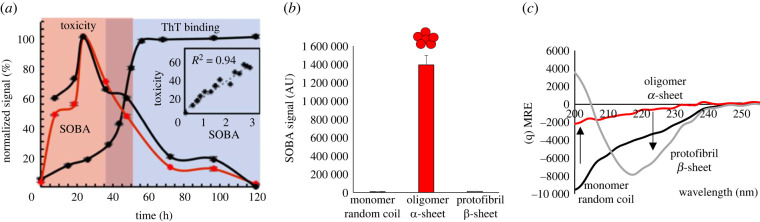
Quantification of soluble Aβ oligomers using SOBA. (*a*) SOBA shows that oligomer content peaks during the late-lag phase of aggregation of Aβ42, and SOBA signal correlates with toxicity, confirming that SOBA has specificity for the toxic, oligomeric form of Aβ. (*b*) Aβ42 spiked into PBS and evaluated by SOBA. Monomer is at beginning of aggregation reaction; toxic oligomers, incubated 24 h; and protofibrils, incubated 120 h (times correspond to (*a*)). (*c*) CD spectra for time points tested on SOBA assay [84]. Figures are taken from [9] and [84].

The soluble oligomer binding assay (SOBA) is a novel ELISA-like assay that detects α-sheet structure in toxic oligomers and is used to quantify toxic α-sheet oligomers in solution [9,62]. By using a *de novo* α-sheet peptide in place of an antibody as the capture agent, SOBA selects for α-sheet structure with high specificity [9,62], providing an indirect readout of α-sheet structure. Also, we note that matched cell toxicity assays with SOBA confirm the strong correlation between α-sheet content as measured by SOBA and toxicity, as inferred from cell viability assays (*R*^2^ = 0.94, figure 9*a*) [9].

SOBA can reproducibly differentiate between toxic Aβ oligomers and the monomeric and protofibrillar/fibrillar forms of the peptide (figure 9*b*) [9,62]. Based on this discrimination between different Aβ conformers (figure 9*b,c*), we explored the detection of toxic oligomers in biological fluids, including cerebrospinal fluid (CSF) and blood. SOBA was able to distinguish between individuals with mild cognitive impairment and moderate to severe AD from non-cognitively impaired controls in both CSF and plasma [84]. The generality of the approach was tested by modifying SOBA to detect α-sheet containing α-synuclein oligomers in samples from patients with PD with excellent discrimination between control and PD cases [84].

## 10. Conclusion

Throughout this review, we have outlined the role of the non-standard α-sheet structure in amyloid aggregation and toxicity. *De novo* α-sheet peptides bind specifically to α-sheet oligomers of a variety of amyloid systems, including both bacterial and mammalian peptides and proteins. This conserved structure reveals a strategy through which to combat amyloid-associated diseases as well as to design diagnostics that can be used to identify a myriad of amyloid diseases in the early stages. This proof of concept has recently been demonstrated for the detection of α-sheet containing oligomers in AD and PD patient samples.

## Data Availability

This article has no additional data.
